# Effect of Leg Extension Angle on Knee Flexion Angle during Swing Phase in Post-Stroke Gait

**DOI:** 10.3390/medicina57111222

**Published:** 2021-11-09

**Authors:** Yuta Matsuzawa, Takasuke Miyazaki, Yasufumi Takeshita, Naoto Higashi, Hiroyuki Hayashi, Sota Araki, Shintaro Nakatsuji, Seiji Fukunaga, Masayuki Kawada, Ryoji Kiyama

**Affiliations:** 1 Doctoral Department, Course of Health Sciences, Graduate School of Health Sciences, Kagoshima University, Kagoshima 890-8544, Japan; k9069833@kadai.jp (Y.M.); k2687318@kadai.jp (Y.T.); k7431050@kadai.jp (S.N.); 2 Miyakonojo Rehabilitation Academy, Miyazaki 885-0062, Japan; 3 Course of Physical Therapy, Faculty of Medicine, School of Health Sciences, Kagoshima University, Kagoshima 890-8544, Japan; k1740552@kadai.jp (S.A.); kawada@health.nop.kagoshima-u.ac.jp (M.K.); kiyama@health.nop.kagoshima-u.ac.jp (R.K.); 4 Master’s Department, Course of Health Sciences, Graduate School of Health Sciences, Kagoshima University, Kagoshima 890-8544, Japan; monkey9.2018@gmail.com (N.H.); 100hapiness@gmail.com (H.H.); 5 Fujimoto General Hospital, Miyazaki 885-0055, Japan; seiji_fukunaga0417@yahoo.co.jp

**Keywords:** leg extension angle, knee flexion angle, stroke, inertial measurement units, gait analysis

## Abstract

*Background and Objectives*: Leg extension angle is important for increasing the propulsion force during gait and is a meaningful indicator for evaluating gait quality in stroke patients. Although leg extension angle during late stance might potentially also affect lower limb kinematics during the swing phase, the relationship between these two remains unclear. This study aimed to investigate the relationship between leg extension angle and knee flexion angle during pre-swing and swing phase in post-stroke gait. *Materials and Methods*: Twenty-nine stroke patients walked along a 16 m walkway at a self-selected speed. Tilt angles and acceleration of pelvis and paretic lower limb segments were measured using inertial measurement units. Leg extension angle, consisting of a line connecting the hip joint with the ankle joint, hip and knee angles, and increments of velocity during pre-swing and swing phase were calculated. Correlation analysis was conducted to examine the relationships between these parameters. Partial correlation analysis adjusted by the Fugl-Meyer assessment-lower limb (FMA-LL) was also performed. *Results*: On the paretic side, leg extension angle was positively correlated with knee flexion angle during the swing phase (*r* = 0.721, *p* < 0.001) and knee flexion angle and increments of velocity during the pre-swing phase (*r* = 0.740–0.846, *p* < 0.001). Partial correlation analysis adjusted by the FMA-LL showed significant correlation between leg extension angle and knee flexion angle during the swing phase (*r* = 0.602, *p* = 0.001) and knee flexion angle and increments of velocity during the pre-swing phase (*r* = 0.655–0.886, *p* < 0.001). *Conclusions*: Leg extension angle affected kinematics during the swing phase in post-stroke gait regardless of the severity of paralysis, and was similar during the pre-swing phase. These results would guide the development of effective gait training programs that enable a safe and efficient gait for stroke patients.

## 1. Introduction

In stroke patients, walking ability is a fundamental function for the activities of daily living and quality of life [1]. Therefore, restoring gait function is a major goal of rehabilitation in stroke patients, and gait training based on appropriate evaluations is necessary to achieve that goal. A post-stroke gait is characterized by the severity of paralysis [2], impaired balance [3], decreased walking speed [4,5], and low propulsion forces on the paretic side [6]. In clinical practice, walking speed is the most common indicator of gait performance post-stroke, and an increase in gait speed is targeted as a goal for gait training in stroke patients. However, Bowden et al. (2006) reported that stroke patients walked depend on the non-paretic lower limb excessively even when achieve sufficient walking speeds [6]. Thus, walking speed does not always represent the function of the paretic limb. It is important to focus not only on walking speed but also lower limb function, kinematics, and the effects of paralysis on gait in stroke rehabilitation.

Propulsion force, the anterior component of ground reaction force (AGRF) during late stance, has been identified as an important factor in post-stroke gait [7] and it is related to walking speed [6]. Recent studies have reported that a critical kinematic variable associated with propulsion force is leg extension angle during late stance [8,9]. Leg extension angle, indicating the lower limb inclination angle during late stance, was shown to be correlated with propulsion force during gait in stroke patients [9,10]. In addition, leg extension angle could be improved by gait training [11,12,13], and alterations in leg extension angle are mainly responsible for an increase in propulsion force following training [14]. Therefore, leg extension angle represents both kinetic and kinematic gait characteristics and would be a feasible and meaningful indicator for evaluating gait quality (such as joint kinematics in clinical practice). In addition, since leg extension angle during late stance contributes to the accumulation of elastic energy by extending the hip flexors and joint capsule [15], it may potentially also affect lower limb kinematics during swing phase (Sw) in post-stroke gait.

During Sw in post-stroke gait, knee flexion angle has been reduced [4,16], which decreases foot clearance [17] and increases gait instability and the risk of falls [18]. Therefore, knee flexion angle during Sw would be a critical indicator of kinematics in post-stroke gait. Factors reported to result in a decrease in knee flexion angle during Sw include the large breaking forces during late stance [19], the excessive activity of the rectus femoris during late stance [20], the lack of push-off power at the ankle [21], and the insufficient hip pull-off during Sw [22]. Consequently, reduced knee flexion during Sw is affected by the gait kinematics during late stance.

Although leg extension angle during late stance and knee flexion during Sw are important factors in post-stroke gait, the relationship between these kinematic parameters remains unclear. The aim of this study was to investigate the relationship between leg extension angle and knee flexion during pre-swing and Sw in stroke patients. We hypothesized that leg extension angle could affect knee flexion during Sw even if the effect of the degree of the lower limb dysfunction is excluded. Elucidation of these interrelationships will help in designing gait training programs for improving knee flexion angle during Sw and walking speed in post-stroke gait.

## 2. Materials and Methods

### 2.1. Participants

Twenty-nine stroke patients (19 male; 16 left hemiparetic; age 59.8 ± 14.0 years; stroke onset 22.5 ± 24.1 months) participated in this cross-sectional study (Table 1). Stroke patients were recruited from inpatient and outpatient departments under the rehabilitation program at Fujimoto General Hospital. The sample size was calculated based on the effect size obtained from a previous report using GPower 3.1.9.7 (Heinrich-Heine-Universität Düsseldorf, Düsseldorf, Germany) which reports that the correlation coefficient between knee flexion angle and plantarflexor muscle activity during gait in stroke patients were 0.68 [23]. Thus, power analysis was performed using the G*Power, *r* = 0.50, α = 0.05, and power (1 − β) = 0.8, indicated that the required sample size was 26. Thus, we accepted that this study had a suitable sample size. Inclusion criteria were as follows: (1) first unilateral stroke, (2) at least three months elapsed since stroke occurring, and (3) the ability to walk independently with or without walking aids (cane and ankle-foot orthosis) for at least 16 m. Exclusion criteria were a history of neurological or orthopedic diseases that could interfere with gait such as cerebellar stroke, joint replacement, and fracture, or cognitive problems that could impair the ability to understand experimental tasks. All participants provided written informed consent prior to initiating the study. This study was approved by the Ethics Committee of Fujimoto General Hospital (Number 177).

Fugl-Meyer assessment-lower limb (FMA-LL, maximum score 34 points) and Brunnstrom recovery stage (BRS) were used to quantify lower limb motor impairment. Modified Ashworth scale (MAS) was used to quantify the lower limb muscle tone. Functional independence measurement (FIM) was used to evaluate performance during activities of daily living.

### 2.2. Gait Measurement

Participants walked 16 m along a straight walkway at a comfortable velocity using the walking aids that they ordinarily used. Gait velocity was calculated from the time taken to walk the middle 10 m using a stopwatch. We measured gait kinematics using five inertial measurement units (IMUs; MTw Awinda, Xsens, Enschede, The Netherlands). The IMUs were attached by elastic belts to the sacrum, bilateral anterior thigh and shank. IMU consist of a 3D gyroscope, 3D accelerometer, and 3D magnetometer. The sampling frequency was 100 Hz. The three-axis acceleration and tilt angles in a global coordinate system were obtained from the magnetic and inertial data using the Kalman filter on the MT manager software (4.7.2, Xsens, Enschede, The Netherlands). The reliability of the software-aided estimation of IMUs has previously been reported [24]. Wherever possible, IMUs were attached along the frontal plane. The vertical direction of the IMU coordinate system was calibrated to align with gravity in a relaxed standing position by the MT manager. In addition, tilt angles and length of thigh and shank were measured during relaxed standing.

### 2.3. Data Analysis

The middle ten gait cycles were analyzed. Low-pass filtering was performed on the angle and the acceleration data measured using IMUs with a 10 Hz and 20 Hz cutoff frequency, respectively. Leg extension angle, hip flexion and extension angle, and knee flexion angle during late stance were calculated from tilt angles and acceleration measured using IMUs during walking, on both the paretic and the non-paretic sides. The measured angles were adjusted by tilt angles of thigh and shank during relaxed standing.

Leg extension angle was defined as the angle made by the laboratory’s vertical axis and a line connecting the lateral malleolus with the greater trochanter in the sagittal plane [25]. Leg extension angle was calculated based on the location of the ankle joint relative to the hip joint in the sagittal plane from the tilt angle measured using the IMU and the vector of the thigh and shank segment coordinated by the segment length, as reported previously [26]. Leg extension angle was adjusted to measure zero at relaxed standing. The maximum leg extension angle during pre-swing was calculated as the representative value.

Hip and knee flexion angles were calculated from the tilt angles of sacrum, thigh, and shank sensors. The maximum hip and knee flexion angle during Sw and maximum hip extension angle during late stance were, respectively, calculated as the representative values. Knee flexion angle at the maximum leg extension angle during pre-swing was also determined.

In addition, the time integral of anterior acceleration measured by the IMU affixed to the sacrum during late stance was calculated as an indicator of the propulsion force. A previous study showed that increments of velocity calculated from acceleration on the sacrum was strongly correlated with the impulse of AGRF during late stance [26]. Data processing was performed using MATLAB R2018b (MathWorks Inc., Natick, MA, USA) mathematical software.

### 2.4. Statistical Analysis

The Shapiro-Wilk test was used to determine whether the data were normally distributed for the following analysis. First, the differences in leg extension angle, hip flexion angle, knee flexion angle, and increments of velocity between the paretic and non-paretic sides were, respectively, compared using paired t-test or Wilcoxon signed-rank test. Next, the association between leg extension angle on the paretic side and hip flexion angle, knee flexion angle, and increments of velocity on the paretic side were investigated using the Pearson’s or Spearman’s rank correlation coefficient. In addition, the mutual association between these kinematic parameters on the paretic side was investigated using the partial correlation analysis after controlling by FMA-LL. SPSS software version 25.0 for Windows (IBM Japan, Tokyo, Japan) was used for statistical analysis. Data are expressed as mean ± standard deviation, and the statistical significance was set at *p* < 0.05.

## 3. Results

The measured gait speed in this study was 0.54 ± 0.30 m/s. The average values of leg extension angle, hip flexion angle, knee flexion angle, and increments of velocity on both the paretic and non-paretic sides are shown in Table 2. On the paretic side, leg extension angle (10.4 ± 7.0°, *p* = 0.006), hip flexion angle (24.8 ± 9.9°, *p* = 0.003), hip extension angle (3.5 ± 8.6°, *p* = 0.007), knee flexion angle (pre-swing, 24.5 ± 14.5°, *p* = 0.001; Sw, 30.9 ± 15.1°, *p* < 0.001), and increments of velocity (0.18 ± 0.09 m/s, *p* < 0.001) were significantly lower than those of the non-paretic side (Table 2).

**Figure 1:**
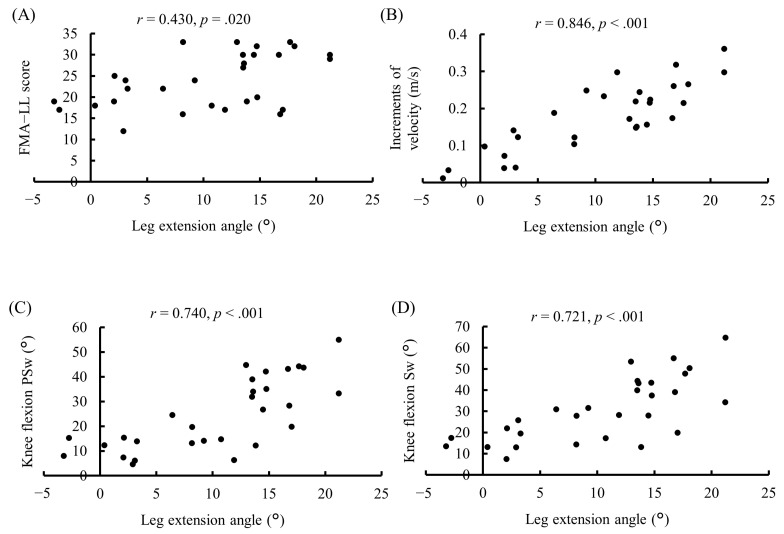
Relationships between leg extension angle and Fugl-Meyer assessment-lower limb (FMA-LL), gait parameters at late stance and swing phase on the paretic side. (**A**) Leg extension angle vs. FMA-LL. (**B**) Leg extension angle vs. knee flexion at pre-swing (PSw). (**C**) Leg extension angle vs. increments of velocity at late stance phase. (**D**) Leg extension angle vs. knee flexion at swing phase (Sw).

The relationships between leg extension angle, FMA-LL, hip flexion angle, hip extension angle, knee flexion angle, and increments of velocity on the paretic side are shown in Table 3 and Figure 1. Leg extension angle showed a significant positive correlation with the FMA-LL (*r* = 0.430, *p* = 0.020, Figure 1a), increments of velocity (*r* = 0.846, *p* < 0.001, Figure 1b), and knee flexion angle during pre-swing (*r* = 0.740, *p* < 0.001, Figure 1c) and Sw (*r* = 0.721, *p* < 0.001, Figure 1d). No significant correlation was observed between leg extension angle and hip flexion angles (*r* = 0.343, *p* = 0.068) and between hip extension angle during late stance and knee flexion angle during Sw (*r* = 0.213, *p* = 0.268) (Table 3).

On the paretic side, partial correlation analysis adjusted by FMA-LL showed that leg extension angle had a significant positive correlation with knee flexion angle during Sw (*r* = 0.602, *p* = 0.001), knee flexion angle during pre-swing (*r* = 0.655, *p* < 0.001), and increments of velocity (*r* = 0.886, *p* < 0.001). No significant correlation was observed between hip extension angle during late stance and knee flexion angle during Sw (*r* = 0.361, *p* = 0.059) (Table 4).

## 4. Discussion

This study investigated the relationships between leg extension angle during late stance and knee flexion angle during the pre-swing phase and Sw in post-stroke gait. Furthermore, the relationship was examined in consideration of the effects of paralysis. The results showed that leg extension angle was significantly associated with knee flexion angle during the pre-swing phase and Sw on the paretic side even after excluding the effects of lower limb motor impairment. This is the first study to demonstrate the association between leg extension angle and knee flexion during Sw in a post-stroke gait.

In a correlation analysis, leg extension angle showed a significant positive association with the increment of velocity during late stance on the paretic side. The increment of velocity during late stance was calculated as the indicator of propulsion force in this study [26]. Our findings were also consistent with previous studies showing that leg extension angle is associated with the AGRF-estimated propulsion force during late stance [8,9]. This study yields evidence to suggest that leg extension angle is a meaningful indicator of the propulsion force in post-stroke gait.

Furthermore, leg extension angle showed a significant positive association with knee flexion angle during the pre-swing phase and Sw on the paretic side. Knee flexion angle during pre-swing has been attributed to the reaction forces passing behind the knee joint. In healthy subjects and children with cerebral palsy, relationships between knee flexion angle during Sw and knee flexion velocity at toe-off has been reported [22,27,28]. Similarly, in stroke patients, knee flexion angle during Sw was shown to be associated with knee flexion velocity at toe-off and ankle plantarflexor moment [21,29]. In addition, sufficient leg extension angle during late stance would be expected to allow hip flexion using elastic energy accumulated by extended hip flexors and the joint capsule. Our results suggest that leg extension angle during late stance could affect knee flexion during the pre-swing phase and Sw. Furthermore, there was no association between hip extension angle during late stance and knee flexion angle during Sw. It was shown that leg extension angle could have a greater influence on knee flexion angle during Sw than hip extension angle during late stance. Therefore, it seems important to improve leg extension angle by gait training in order to maintain sufficient knee flexion angle and foot clearance during Sw.

Partial correlation analysis adjusted by FMA-LL, leg extension angle was still significantly related to knee flexion angle during pre-swing and Sw on the paretic side. These results showed that leg extension angle could exert a significant effect on knee flexion angle during the pre-swing phase and Sw, regardless of the degree of paralysis. FMA-LL has been shown to be correlated with cadence and step length [30]. Although FMA-LL is an important factor in post-stroke gait, it was not the sole determinant of knee flexion angle during Sw in this study. Therefore, stroke rehabilitation to improve walking ability would require direct gait intervention in addition to training aimed at improving paralysis.

In addition, partial correlation analysis showed knee flexion angle during Sw was significantly associated with knee flexion angle during the pre-swing phase. Several studies have shown that knee flexion should begin in terminal stance (that is, during single support phase of gait) and that it can reach its normal swing peak only if knee flexion velocity during the pre-swing phase is adequate [28,31]. Furthermore, it has previously been reported that knee flexion angle during Sw increases when stroke patients receive assistive knee flexion torque at toe-off [32]. Thus, it seems important to secure knee flexion angle during pre-swing in order to attain sufficient knee flexion angle during Sw. This study also showed that knee flexion angle during pre-swing was strongly associated with leg extension angle. Considering these results, leg extension angle could allow sufficient knee flexion during pre-swing and affect knee flexion during Sw in post-stroke gait. Therefore, a secure leg extension angle with knee flexion during the pre-swing phase seems important for safe and efficient gait in patients with stroke.

This study had several limitations. First, previous studies have reported the effect of ankle plantarflexor moment and muscle activity on leg extension angle and propulsion force in the paretic lower limb [10,33,34]. However, we cannot conduct multiple analyses considering that the ankle joint motion was not analyzed in this study due to the effects of walking aid (such as foot orthosis). Second, most participants in this study used walking aids such as ankle-foot orthosis or T-canes, which might affect leg extension angle and knee flexion angle. The inconsistent effects of ankle-foot orthosis on knee flexion angle during Sw have reported [35,36]. Therefore, additional studies are needed to clarify the relationships between gait kinematics during late stance and during Sw.

## 5. Conclusions

Our results established a positive association between leg extension angle and knee flexion angle during Sw as well with the propulsive force during late stance, even when the effects of lower limb motor impairment were excluded. Similar results were shown during the pre-swing phase. Leg extension angle could reflect the kinematics and kinetics during the pre-swing phase and Sw in post-stroke gait, and it can be a meaningful indicator for estimating gait quality during gait training. These results could inform the design of effective gait training programs aimed at achieving a safe and efficient gait in stroke patients.

## Figures and Tables

**Table 1:** Clinical characteristics of subjects.

Variable	Patients (*n* = 29)
Age (years)	59.8 ± 14.0
Sex (*n*): male/female	19/10
Height (cm)	162.7 ± 8.0
Weight (kg)	65.6 ± 11.1
Disease (*n*): hemorrhage/infarction	17/12
Time post-stroke (months)	22.5 ± 24.1
Affected side (*n*): right/left	13/16
T-cane (*n*)	26
Ankle foot orthosis (*n*)	24 (DF free and PF limitation 17, DF free and PF braking 7)
BRS (*n*)	III: 10 IV: 8 V: 7 VI: 4
FMA-LL (points)	23.9 ± 6.4
MAS knee flexion muscles (*n*)	0: 16 1: 8 1+: 4 2: 1
MAS knee extension muscles (*n*)	0: 12 1: 11 1+: 5 2: 1
MAS ankle PF muscles (*n*)	0: 4 1: 5 1+: 11 2: 7 3: 2
FIM walk (*n*)	5: 4 6: 23 7: 2

BRS: Brunnstrom recovery stage; FMA-LL: Fugl-Meyer assessment-lower limb; MAS: modified Ashworth scale; FIM: functional independence measurement; DF: dorsiflexion; PF: plantarflexion.

**Table 2:** Joint angles and increments of velocity calculated from the acceleration of pelvis during gait.

Variable	Paretic	Non-Paretic	*p*-Value
Late stance phase			
Leg extension angle (°)	10.4 ± 7.0	14.3 ± 4.8	0.006
Hip extension (°)	3.5 ± 8.6	8.6 ± 7.2	0.007
Knee flexion during PSw (°)	24.5 ± 14.5	35.0 ± 11.4	0.001
Increments of velocity (m/s)	0.18 ± 0.09	0.33 ± 0.11	<0.001
Swing phase			
Peak hip flexion (°)	24.8 ± 9.9	29.8 ± 6.5	0.003
Peak knee flexion (°)	30.9 ± 15.1	55.6 ± 10.0	<0.001

PSw: pre-swing.

**Table 3:** The correlations between leg extension angle and gait parameters at late stance and swing phase on the paretic side.

Variable	1	2	3	4	5	6	7
Lower limb motor function							
1. FMA-LL	-	0.430 *	0.004	0.695 **	0.121	0.504 **	0.695 **
Late stance phase							
2. Leg extension angle		-	0.629 **	0.740 **	0.846 **	0.343	0.721 **
3. Hip extension			-	0.204	0.702 **	−0.204	0.213
4. Knee flexion (PSw)				-	0.436 *	0.533 **	0.513 **
5. Increments of velocity					-	0.184	0.513 **
Swing phase							
6. Hip flexion						-	0.700 **
7. Knee flexion							-

* *p* < 0.05, ** *p* < 0.01. FMA-LL: Fugl-Meyer assessment-lower limb; PSw: pre-swing.

**Table 4:** Partial correlations between leg extension angle and gait parameters at late stance and swing phase on the paretic side after controlling by FMA-LL.

Variable	1	2	3	4	5	6
Late stance phase						
1. Leg extension angle	-	0.754 **	0.655 **	0.886 **	0.079	0.602 **
2. Hip extension		-	0.299	0.724 **	−0.217	0.361
3. Knee flexion (PSw)			-	0.536 **	0.300	0.840 **
4. Increments of velocity				-	0.098	0.536 **
Swing phase						
5. Hip flexion					-	0.500 **
6. Knee flexion (Sw)						-

** *p* < 0.01. FMA-LL: Fugl-Meyer assessment-lower limb; PSw: pre-swing; Sw: swing phase.

## Data Availability

The data used to support the findings of current study are available from the corresponding author upon request.
